# Reduced Plasma Levels of α-Klotho and Their Correlation With Klotho Polymorphisms in Elderly Patients With Major Depressive Disorders

**DOI:** 10.3389/fpsyt.2021.682691

**Published:** 2021-10-13

**Authors:** Xiang Gao, Zuoli Sun, Guangwei Ma, Yuhong Li, Min Liu, Guofu Zhang, Hong Xu, Yane Gao, Jixuan Zhou, Qi Deng, Rena Li

**Affiliations:** ^1^Laboratory of Brain Disorders, Ministry of Science and Technology, Beijing Institute of Brain Disorders, Collaborative Innovation Center for Brain Disorders, Capital Medical University, Beijing, China; ^2^Beijing Key Laboratory of Mental Disorders, Beijing Anding Hospital, Capital Medical University, Beijing, China

**Keywords:** α-Klotho, plasma level, polymorphism, depression, rs9315202

## Abstract

**Background:** Recent literature suggests that α-Klotho, a widely recognized anti-aging protein, is involved in longevity as well as in many diseases, including Alzheimer's disease, and depression. Although the *Klotho* gene encodes α-Klotho, a single transmembrane protein with intracellular and extracellular domains, the relationship between *Klotho* gene polymorphism and circulating α-Klotho levels in patients with major depressive disorder (MDD) is not clear.

**Methods:** A total of 144 MDD patients and 112 age-matched healthy controls were included in this study. The *Klotho* genetic polymorphisms (rs9536314, rs9527025, and rs9315202) and plasma α-Klotho levels were measured by PCR and ELISA, respectively. The severity of depressive symptoms was estimated using the Hamilton Depression Scale (HAMD).

**Results:** We found a significantly lower level of plasma α-Klotho in the MDD patients than in controls. Among them, only elderly MDD patients (first episode) showed significantly lower α-Klotho levels than the age-matched controls, while elderly recurrent and young MDD patients showed no difference in plasma α-Klotho levels from age-matched controls. The young MDD group showed a significantly earlier onset age, higher plasma α-Klotho levels, and lower HAMD scores than those in the elderly MDD group. While the plasma α-Klotho levels were higher in rs9315202 T alleles carrier regardless age or sex, the rs9315202 T allele was negatively correlated with disease severity only in the elderly MDD patients.

**Conclusion:** The results of our study showed that only elderly MDD patients showed a decrease in plasma α-Klotho levels along with an increase in disease severity as well as an association with the number of rs9315202 T alleles, and not young MDD patients compared to age-matched controls. Our data suggest that circulating α-Klotho levels combined with *Klotho* genetic polymorphisms are important in elderly MDD patients, particularly carriers of the *Klotho* gene rs9315202 T allele.

## Introduction

α-Klotho, an anti-aging protein, is known for its aging-related functions in longevity. Reduced expression of the *Klotho* gene or α-Klotho protein level are associated with a shorter lifespan and risk of neurodegenerative diseases, as well as accelerated aging and premature morbidity and mortality (1). α-Klotho protein is a single-pass type I transmembrane protein with three domains: a short intracellular domain (~60 amino-acids), a transmembrane domain, and a large extracellular domain (~950 amino-acids). The full-length α-Klotho protein (~140 kD) is a substrate for several enzymes (2, 3), and the cleaved extracellular domain (~130 kD) enters the blood, urine, and cerebrospinal fluid as a soluble α-Klotho protein (4, 5). While the full-length membrane-bound α-Klotho protein is known as a co-receptor for fibroblast growth factor-23 as well as playing a role in regulating the excretion of phosphate and the synthesis of active vitamin D (6–8), the soluble α-Klotho protein acts as a hormonal factor as well as an enzyme in regulating growth factor signaling (9–11), oxidative stress (12), and ion channels and transporters (13–16).

As an anti-aging protein, α-Klotho is widely expressed throughout the body and is most strongly expressed in the brain (17). Recent literature suggest that the level of α-Klotho is regulated by chronic stress and depression. For example, a study showed that women under a higher level of stress have a significant age-dependent reduction of plasma α-Klotho compared with women under low stress, while the lower level of α-Klotho is also associated with more severe depressive symptoms (18). In addition, treatment for MDD might upregulate soluble α-Klotho protein levels in elderly depressive patients. For example, electroconvulsive therapy (ECT) results in a significant increase in cerebrospinal fluid (CSF) α-Klotho levels, which are positively correlated with the frequency of ECT treatment (19). However, other studies failed to confirmed these findings and showed no difference in soluble α-Klotho levels before and after ECT or antidepressant treatment in patients with MDD (20).

Previous studies have suggested that *Klotho* gene polymorphisms are also associated with mental disorders. KL-VS is the most well-studied α-Klotho haplotype and consists of six sequence variants, which was in perfect linkage disequilibrium (LD) in the haplotype. Two of variants are located in exon 2 and result in the amino acid substitutions, F352V (rs9536314) and C370S (rs9527025) (21). In the treatment of late-life MDD, patients who carry *Klotho* genetic polymorphism KL-VS (rs9536314) homozygotes respond poorly to selective serotonin reuptake inhibitors (SSRIs) compared to non-carriers after 6 months of treatment (22). Moreover, it has been suggested that KL-VS various (rs9536314/rs9527025) affect the trafficking and catalytic activity of α-Klotho protein (21), and KL-VS heterozygous carriers have higher serum α-Klotho levels than non-carriers (23). While very limited studies investigated the relationship of rs9315202 with aging and psychiatric stress-related DNA methylation, a recent postmortem PTSD brain study showed that the rs9315202 is associated with a reduction of *Klotho* expression via DNA methylation in elderly with psychiatric stress as PTSD (24). There is another clinical study with Caucasians suggested that *Klotho* SNP rs9315202 might predict a consistent correlation between psychiatric stress and cell aging since the severity of PTSD was more strongly associated with advanced epigenetic age in those carried two copies of the minor allele (25). In addition, it has been hypothesized that the potential effect of rs9315202 on *Klotho* gene expression may be through transcription regulation, since it is located at a potential enhancer (enh51509) of the *Klotho* gene (Enhancer DB) (26).

*Since Klotho* is closely related to age, it is hypothesized that *Klotho* polymorphism may exert their biological effect such as aging and longevity in specific time windows. This hypothesis is supported by the KL-VS heterozygous genotype only favorable for survival in old people, not in centenarian (27) or young controls (21).While limited investigations focused on the anti-aging factor of *Klotho* and treatment response in MDD, whether the *Klotho* SNPs distribution contribute α-Klotho protein expression and disease severity in MDD patients of different ages is still unknown. In this study, we detected the *Klotho* polymorphism (rs9536314 and rs9315202) and α-Klotho plasma concentration in young and elderly MDD patients and healthy controls, then analyzed the potential clinical association and significance.

## Materials and Methods

### Participants

Samples from 144 MDD patients and 112 age-matched healthy controls were selected from the Beijing Biobank of Clinical Resources-Mental Disorders. Participants in two age groups were included in the study: young (*n* = 71, 30–40 years) and elderly (*n* = 73, >65 years). All patients were diagnosed previously by two experienced psychiatrists according to the Diagnostic and Statistical Manual of Mental Disorders-IV and their personal information, disease state, age of onset, and Hamilton Depression Scale (HAMD) scores were collected as a standard protocol for the Biobank. Genomic DNA was extracted from all participants for SNP analysis and the α-Klotho levels were measured in the plasma of all participants. The patients were further divided into a first episode group and a recurrent group. The exclusion criteria included a history of substance abuse or dependency and any neurological or psychiatric disorder. The study was approved by the Ethics Committee of Beijing Anding Hospital and was in accordance with the latest version of the Declaration of Helsinki. All participants provided written informed consent to participate in this study.

### Measurement of Plasma α-Klotho Levels

Blood samples were collected from both MDD patients and healthy controls in EDTA tubes and plasma was isolated by centrifugation at 3,000 rpm for 10 min and stored at −80°C until analysis. Concentrations of plasma α-Klotho were assessed using solid-phase sandwich enzyme-linked immunosorbent assays following the manufacturer's instructions (Immuno-Biological Laboratories, Takasaki, Japan). In brief, 100-μl plasma samples per well of a pre-coated 96-well plate were incubated for 60 min at room temperature before washing 4 times with buffer. Then, the samples were incubated with 100 μl antibody for 30 min at room temperature followed by 5 washes. The chromogen (100 μl) was added to the wells and incubated for 30 min in the dark before adding 100 μl Stop solution to stop the reaction. A SpectraMax i3x reader (Molecular Devices, San Jose, CA) at 450 nm was used for data acquisition. The sensitivity of the ELISA kit was 6.15 pg/ml and the coefficient of variation was <10%.

### *Klotho* SNP Assays

DNA was extracted from leukocytes using the Miniprep System (Promega, Madison, USA). The concentration of DNA was measured by using Nanodrop 2000 (Thermo Scientific, Waltham, USA) to ensure that adequate amounts of high-quality genomic DNA had been extracted. A primer pool containing three SNPs (rs9536314, rs9527025, and rs9315202) was designed and synthesized, and then the target SNPs were amplified by two-step PCR and an Illumina-compatible library was prepared. After the first round of PCR reaction, the PCR products were detected by 1% agarose gel electrophoresis to determine the correct size of the products. The PCR products were purified using AMPureXP beads (Beckman-Coulter, CA, USA). Then the second round of PCR reaction was performed using the first round PCR products as templates to obtain the sequenced library with molecular tags as previously described (28). After the final PCR products were recovered and mixed equally, paired-end sequencing of the library was performed on HiSeqXTen sequencers (Illumina, San Diego, CA). Prinseq-lite (v 0.20.3) software was used to filter the raw data, and the remaining clean data were mapped to the reference genome using BWA (v 0.7.13-r1126) software, and the genotype results of the target site were calculated according to the comparison results.

### Statistical Analysis

All experimental data were entered into a database in SPSS 26.0 software (SPSS Inc., Chicago, Illinois, USA). All quantitative data accord with normal distribution by Kolmogorov-Smirnov test, and are presented as the mean ± SD. Comparisons of the data (plasma α-Klotho levels and HAMD scores) between groups were tested using the unpaired *t*-test or ordinary one-way ANOVA. Chi-squared test was used to detect and verify whether the genotype frequency distributions in participants was consistent with Hardy-Weinberg equilibrium (HWE). Chi-squared test was also used to detect and verify whether there is difference in the genotype frequency distributions (Recessive, Dominant, Additive, Genotype and Allele models) between two groups. Pearson correlations were used to examine the correlation between age and plasma α-Klotho levels. Age× genotype interaction model was used to investigate whether there was a significant rs9315202dominant × age e?ect on the plasma α-Klotho levels in all participants. Multiple linear regression analysis was used to analyze the correlation between plasma α-Klotho levels and the rs9315202 T allele. The level of significance was set at *p* < 0.05. However, the corrected *p* value was set at 0.0167 considering the Bonferroni multiple testing. A power calculation for the main results was performed. Group sample sizes of 32 (healthy controls) and 28 (first-episode patients) achieve 78.975% (>75%) power to reject the null hypothesis of equal means (Plasma α-Klotho levels) when the population mean difference is μ1–μ2 = 581.1–443.9 = 137.3 with standard deviations of 172.2 for group 1 and 201.4 for group 2, and with a significance level (alpha) of 0.050 using a two-sided two-sample unequal-variance *t*-test. All significance tests were two-tailed.

## Results

### Plasma α-Klotho Concentration Is Reduced in the Elderly, and Not in Young MDD Patients Compared to Age-Matched Controls

The demographic information of all MDD patients as well as controls is shown in Table 1. While we found a significantly lower level of plasma α-Klotho in the MDD patients than in the control group (714.767 ± 313.075 vs. 808.660 ± 312.629 pg/ml, *P* = 0.018; Supplementary Table 1), further analysis showed that the lower level was limited to the elderly first-episode MDD group compared to the age-matched elderly control group (443.871 ± 201.352 vs. 581.125 ± 172.242 pg/ml, *P* = 0.006), but not the recurrent elderly MDD group (586.147 ± 227.622 vs. 581.125 ± 172.242 pg/ml, *P* = 0.917) (Figure 1A). Furthermore, our data showed no difference of plasma α-Klotho levels between young MDD and age-matched controls, regardless disease episode (Figure 1A). In addition, while all participants showed a significant correlation between plasma α-Klotho concentration and age (*P* < 0.001, *r* = −0.564; Supplementary Table 2), the young MDD groups showed significant higher plasma α-Klotho levels than the matched elderly MDD groups (Figure 1A). It is also interesting that the young MDD groups had less severe clinical symptoms as shown by lower HAMD scores (Figure 1B), and an earlier onset of the disease (Table 1) than the matched elderly MDD groups.

**Table 1:** Demographic and clinical data in elderly and young participants (*mean* ± *SD*).

**Characteristics**	**Elderly**			**Young**		
	**HC**	**FEP**	**RP**	**HC**	**FEP**	**RP**
N	32	28	45	80	35	36
Female (%)	19 (59.4)	18 (64.3)	31 (68.9)	41 (51.2)	23 (65.7)	18 (50.0)
Age	63.97 (3.04)	67.04 (5.78)	67.69 (4.49)	34.83 (3.44)**^###^**	34.06 (3.12)**^###^**	34.89 (3.50)**^###^**
Onset age	NA	66.11 (5.70)	63.49 (3.75)	NA	33.91 (4.17)**^###^**	25.90 (6.80)**^###^**
Plasma α-Klotho levels, pg/ml	581.125 (172.242)	443.871 (201.352)^**^	586.147 (227.622)	899.674 (310.364)**^###^**	942.630 (279.935)**^###^**	864.705 (270.012)**^###^**
HAMD scores	NA	27.07 (7.06)	24.71 (7.41)	NA	9.63 (6.77)**^###^**	11.75 (8.17)**^###^**

*HC, healthy controls; FEP, first-episode patients; RP, recurrent patients. Significant difference between controls and patients,*

** *P < 0.01; significant difference between elderly and young group,*

### *P < 0.001*.

**Figure 1:**
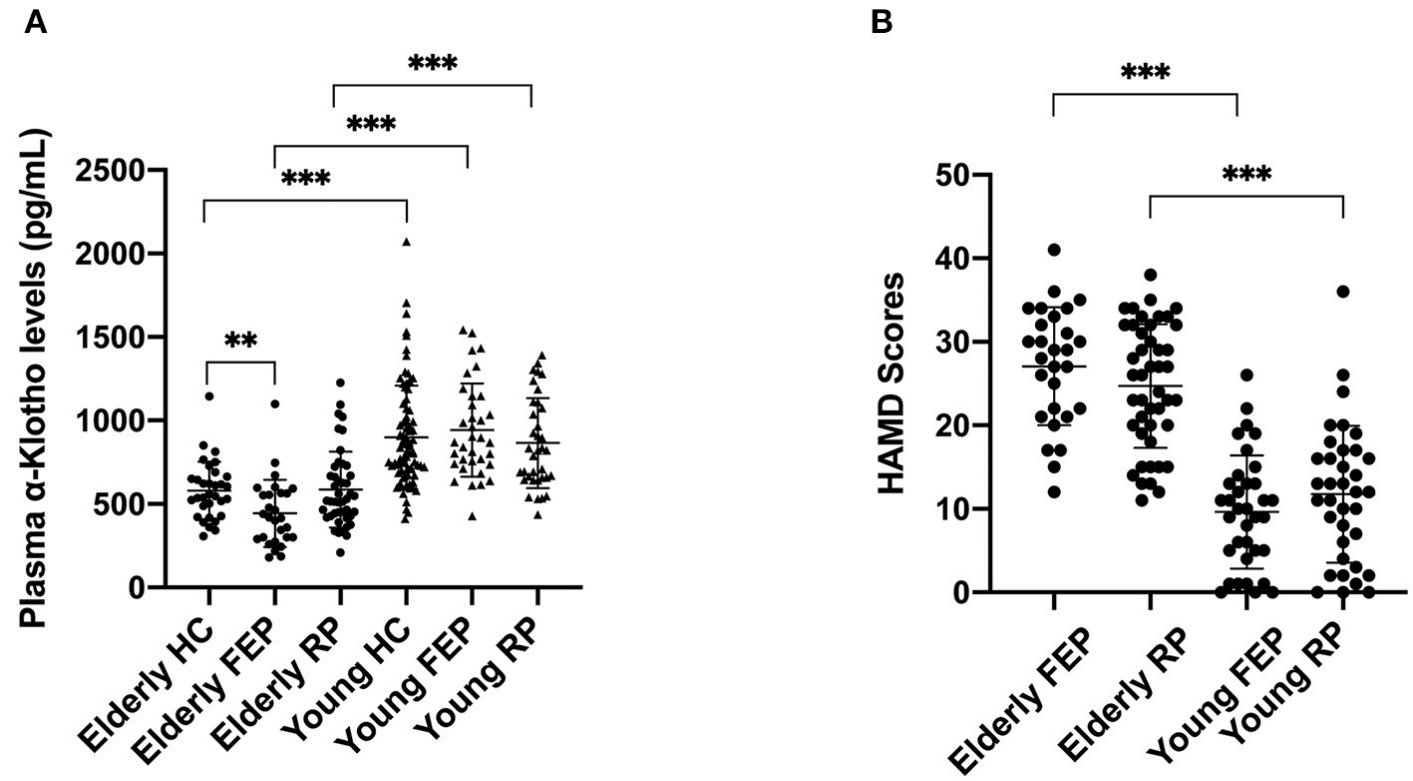
Plasma α-Klotho levels **(A)** and HAMD scores **(B)** in participants. HC, healthy controls; FEP, first episode patients; RP, recurrent patients; ^**^P < 0.01, ^***^P < 0.001.

### Genotyping and Allele Distributions of SNP rs9315202 in MDD Patients and Matched Controls

**Figure 2:**
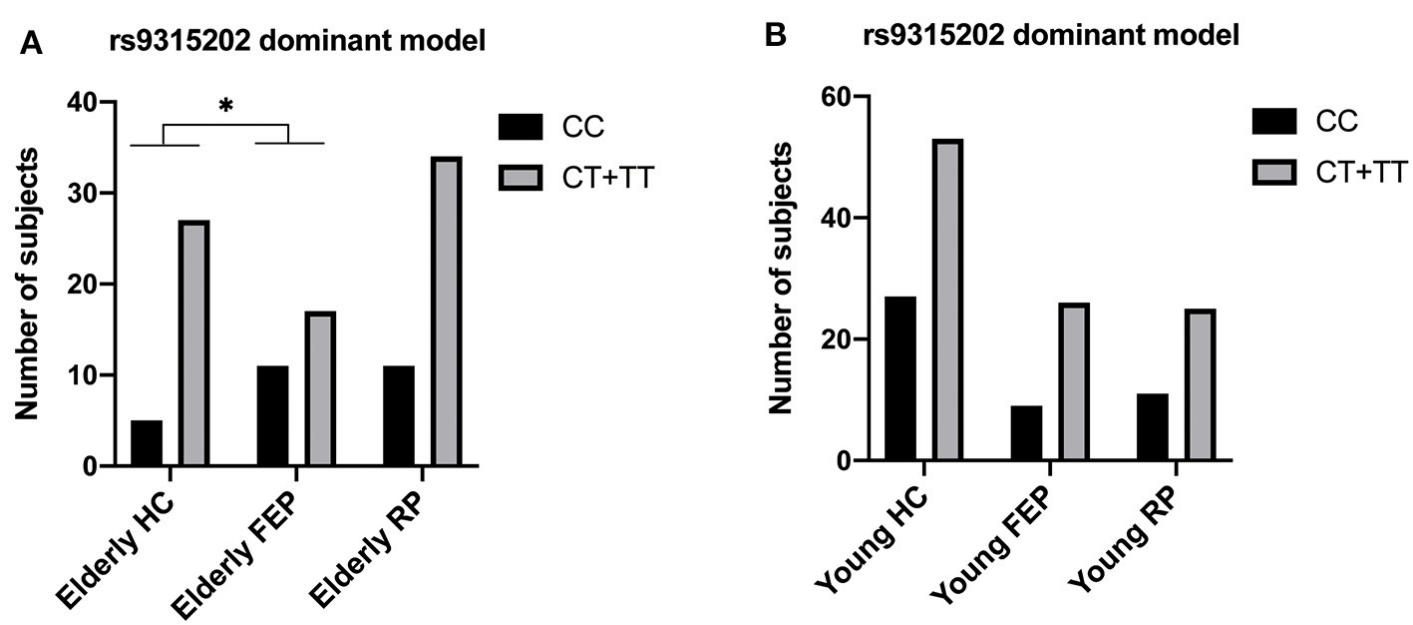
Genotyping and dominant model distributions of SNP rs9315202 in the elderly **(A)** and young **(B)** participants. HC, healthy controls; FEP, first episode patients; RP, recurrent patients; ^*^P < 0.05.

The *Klotho* genotype was distributed in HWE (*P* > 0.05). Among the 256 participants, only two (an elderly MDD patient and a young healthy control) carried KL-VS (rs9536314/rs9527025) loci. Since the minimum allele frequency of rs9536314/rs9527025 was <0.005, the KL-VS (rs9536314) data were excluded (Supplementary Tables 4–5). Interestingly, the distribution frequency of rs9315202 T allele is significant lower in the elderly first-episode patients than the elderly control group. A significant difference in the distribution frequency of the rs9315202 dominant model (CC vs. CT + TT) was found between the elderly first episode MDD group and the elderly control group (*P* = 0.039), while the elderly recurrent MDD group had a distribution frequency similar to the elderly controls (*P* = 0.347; Table 2 and Figure 2A). No significant difference in distribution of this model (CC vs. CT + TT) was found between the young MDD and young control groups (Figure 2B and Supplementary Table 3).

**Table 2:** Genotyping and allele distributions of SNP rs9315202 in the elderly participants.

**Groups**	**rs9315202 genotype models**											**HWE**
	**Recessive**		**Dominant**		**Additive**		**Genotype**			**Allele**		
	**CC+CT**	**TT**	**CC**	**CT+TT**	**CC**	**TT**	**CC**	**CT**	**TT**	**C**	**T**	
Elderly HC	26	6	5	27	5	6	5	21	6	31	33	0.21
Elderly FEP	21	7	11	17	11	7	11	10	7	32	24	0.36
Elderly RP	33	12	11	34	11	12	11	22	12	44	46	0.99
χ^2^												
χ^2^_ElderlyFEP_	0.344		4.275		0.192		5.990			0.908		
χ^2^_ElderlyRP_	0.654		0.884		0.017		2.139			0.003		
***P***												
*P*_ElderlyFEP_	0.558		**0.039**		0.661		**0.050**			0.341		
*P*_ElderlyRP_	0.419		0.347		0.897		0.343			0.956		

*HC, healthy controls; FEP, first-episode patients; RP, recurrent patients; data were analyzed by Pearson's χ^2^ test.*

*Bold values are used to represent P values ≤ 0.05*.

### Relationship Between the rs9315202 Genotype and Plasma a-*Klotho* Concentration

**Figure 3:**
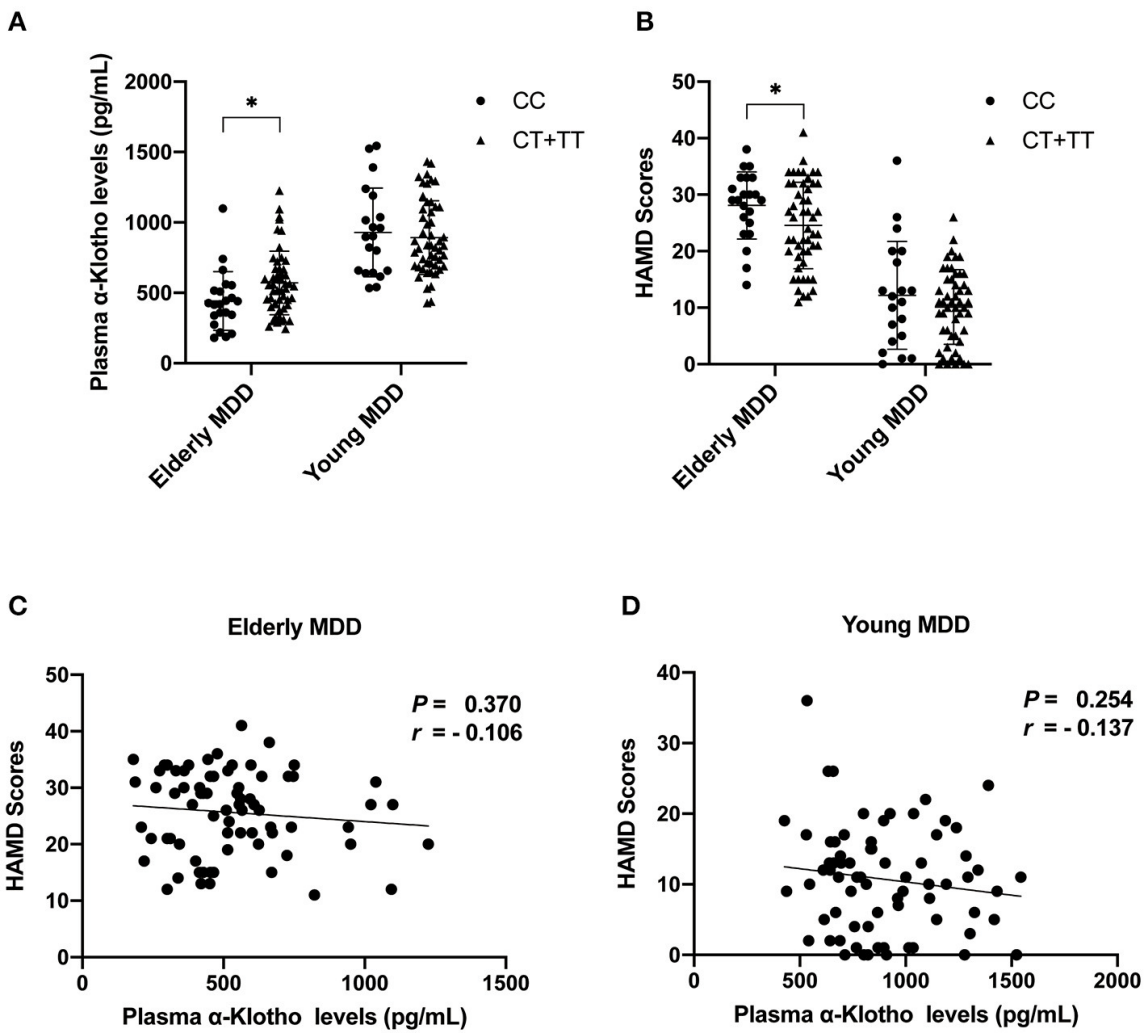
Plasma α-Klotho levels **(A)** and HAMD scores **(B)** in MDD patients carrying *Klotho* polymorphism rs9315202, and HAMD scores vs. α-Klotho levels in elderly **(C)** and young **(D)** patients. MDD, major depressive disorders; ^*^P < 0.05.

As we found a significant difference in plasma α-Klotho levels as well as *Klotho* rs9315202 genotype between the elderly first-episode MDD and age-matched healthy control groups, to understand the impact of *Klotho* rs9315202 genotypes on plasma α-Klotho concentration, we divided the participants into two groups according to genotype (CC vs. CT + TT) (Table 3). A significantly lower plasma level of α-Klotho was found in the CC genotype carriers than in the CT + TT genotype carriers among all participants regardless age and disease (686.803 ± 300.067 vs. 783.917 ± 318.416 pg/ml, *P* = 0.025; Table 3). In the healthy controls, a lower plasma α-Klotho level was found in the young CC carriers than in the age-matched CT+TT carriers (749.706 ± 182.738 vs. 976.072 ± 334.691 pg/ml, *P* < 0.001), but not in elderly controls (455.062 ± 114.755 vs. 604.470 ± 172.408 pg/ml, *P* = 0.074; Table 3). In the MDD patients, only the elderly patients showed a significantly lower plasma level of α-Klotho (442.118 ± 207.958 vs. 570.165 ± 226.433 pg/ml, *P* = 0.026) in the CC genotype carriers than in the CT + TT carriers, while no association between plasma α-Klotho and genotype was found in the young MDD patients, although an association was found in young healthy controls (Table 3). Age-dependent relationships between the rs9315202 genotype and plasma a-Klotho concentration as well as HAMD scores in the young and elderly MDD groups were also found (Figure 3). Our data showed that in the elderly MDD patients, the disease severity was significantly different between the CC and CT + TT carriers (28.09 ± 5.94 vs. 24.55 ± 7.64, *P* < 0.05; Figure 3B). However, there is no significant association between HAMD scores and plasma α-Klotho levels in both elderly and young MDD patients (Figures 3C,D). The rs9315202 dominant model × age did not predict plasma α-Klotho concentration, but the age evidenced a significant main effect (*P* = 0.012; Table 4). In addition, the multiple linear regression model was used to control for the effects of variables significantly associated with plasma α-Klotho concentration (Table 5). In all participants, age was used in the first model 1, *F* = 118.385, *P* < 0.001, with an *R*^2^ of 0.318. Adding the rs9315202 T allele improved model 2, *F* = 69.884, *P* < 0.001, with an *R*^2^ of 0.356. Adding rs9315202 T allele and sex improved model 3, *F* = 50.537, *P* < 0.001, with an *R*^2^ of 0.376. For all 256 participants, age was the main factor negatively affecting plasma α-Klotho concentration, while sex and the rs9315202 T allele had a weak positive effect.

**Table 3:** Plasma α-Klotho levels (pg/ml) according to the distribution of rs9315202 dominant model (*mean* ± *SD*).

**Groups**	***N***	**Plasma α-Klotho levels**		***t***	***P***
		**CC**	**CT+TT**		
All participants	256	686.803 (300.067)	783.917 (318.416)	−2.249	**0.025**
Elderly HC	32	455.062 (114.755)	604.470 (172.408)	−1.850	0.074
Elderly MDD	73	442.118 (207.958)	570.165 (226.433)	−2.270	**0.026**
Young HC	80	749.706 (182.738)	976.072 (334.691)	−3.911	**<0.001**
Young MDD	71	928.973 (314.427)	892.980 (261.795)	0.492	0.624

*HC, healthy controls; MDD, major depressive disorder.*

*Bold values are used to represent P values ≤ 0.05*.

**Table 4:** Regression results from models with significant interactive effects of rs9315202 dominant model and age on plasma α-Klotho concentration (*n* = 256).

**Variable**	***Beta***	***SE***	***P***
**Covariates**			
Age	−0.747	5.331	**0.012**
Sex	0.013	45.032	0.851
Onset	−0.017	4.642	0.950
rs9315202 dominant model	−0.257	157.094	0.259
rs9315202 dominant model × age	0.389	2.833	0.120

*Bold values are used to represent P values ≤ 0.05*.

**Table 5:** Multiple linear regression model of plasma α-Klotho concentration in total participants (*n* = 256).

**Models**	**Model summary**			**Multiple regression weights**		
	***R*** **^2^**	***F*** **(df)**	***P***	***B***	***Beta***	***P***
Model 1	0.318	118.385 (1, 254)	**<0.001**			
Age				−11.035	−0.564	**<0.001**
Model 2	0.356	69.884 (1, 253)	**<0.001**			
Age				−11.359	−0.580	**<0.001**
rs9315202 T allele				90.218	0.196	**<0.001**
Model 3	0.376	50.537 (1, 252)	**<0.001**			
Age				−11.679	−0.597	**<0.001**
rs9315202 T allele				92.957	0.201	**<0.001**
Sex				90.628	0.142	**0.005**

*Bold values are used to represent P values ≤ 0.05*.

## Discussion

Recent literature suggests that the level of α-Klotho is regulated by chronic stress and depression (18), and polymorphisms of the *Klotho* gene affect the plasma level of α-Klotho in healthy individuals (23). To investigate whether the plasma level of α-Klotho and SNPs are associated with MDD, we examined both of them in MDD patients and healthy controls. While extensive reports have shown that α-Klotho levels decline with age, we found that elderly MDD patients had even lower levels than age-matched controls (Figure 1A). However, such a difference in plasma α-Klotho levels did not exist in young MDD patients and matched controls. Our data suggested that circulating α-Klotho might be involved in MDD in elderly, but not young patients. Previous studies have demonstrated the age-dependent changes of circulating α-Klotho concentrations, including those in the plasma (29) as well as in the CSF (30).

Moreover, as a previous study suggested that a lower level of α-Klotho is associated with more severe stress-related depressive symptoms in healthy individuals (18), we speculated that the decrease of plasma α-Klotho with aging may contribute to the aggravation of depressive symptoms in MDD patients. Besides, MDD is a highly heterogeneity disease, as exemplified by early vs. late onset, first episode vs. recurrent MDD, and other subtypes (31). Previous studies have shown that patients with early-onset depression (EOD) and late-onset depression (LOD) have different symptomatology and clinical characteristics (32, 33) as well as biological processes (32, 33). For example, the serum levels of interleukin-10 in LOD patients are higher than in controls or EOD patients (34), and the increased plasma C-reactive protein (CRP) levels are more closely associated with LOD than EOD patients (35). In the current study, we noticed that the onset of age in young MDD patients is significantly earlier than that in elderly MDD patients (Table 1). Whether the difference in plasma α-Klotho levels between young and elderly MDD patients is due to the difference in onset ages or the patients age still need further investigation with age-matched EOD and LOD patients.

In addition to age, soluble α-Klotho concentration has also been associated with the type and status of disease (36–38). For example, soluble α-Klotho levels significantly decrease with the severity of chronic kidney disease (39). Furthermore, the concentration of plasma α-Klotho is negatively correlated with the severity of progression of cerebral small vessel disease in patients with acute ischemic stroke (40). An association between circulating α-Klotho levels and disease severity has also been found in patients with cerebral deep white-matter lesions and impaired cognitive function (41). Moreover, the level of plasma α-Klotho might also reflect the level of psychosocial stress as women under high-stress condition expressed significant reduction in plasma α-Klotho compared with the women under low-stress category (18). While α-Klotho is associated with aging and MDD is also closely related to aging (19, 20, 22), vascular disease (42, 43), stress (25, 44, 45) and cognitive decline (46–49), we studies the α-Klotho in young and old MDD patients with age-matched controls. Interestingly, we noted a reduced plasma α-Klotho level in elderly first-episode MDD patients, not in elderly patients with recurrent MDD, compared to elderly controls (Table 1; Figure 1A). We found no significant difference in plasma α-Klotho between the young MDD group and age matched controls (Table 1; Figure 1A). These results in young participants is consistent with a previous study which found no significant difference in plasma levels of α-Klotho between MDD patients and healthy controls at an average age of 45 years (20).

Medical treatment may be another important reason for the change of soluble α-Klotho concentration. Hoyer et al. found that ECT elevates the CSF-soluble α-Klotho levels in patients with MDD (19), suggested that treatment may affect the soluble α-Klotho levels. However, Sartorius et al. found no group differences between the baseline soluble α-Klotho of MDD patients and controls and between pre- and post-treatment in MDD patients treated either with ECT or antidepressants (20). In general, there is no consistent conclusion about the effect of treatment on soluble α-Klotho levels (including plasma and CSF). Compared with the elderly recurrent MDD patients, the elderly first- MDD patients received no or little medication, which might represent antidepressant treatment affects plasma α-Klotho levels. However, such an argument is not supported in young MDD patients since there was no difference in plasma α-Klotho between the first-episode and recurrent MDD groups (Figure 1). Instead, the first-episode young MDD patients had a higher α-Klotho level than that in the recurrent patients (Table 1), but the difference did not reach statistical significance. Interestingly, the first-episode young MDD group had a significantly later onset age than the age-matched recurrent MDD group (Table 1). Given the earlier onset age in young MDD than elderly MDD found in our study, whether the plasma α-Klotho levels are associated more with the onset age rather than the medication requires further investigation.

Mounting evidence link the polymorphism of the *Klotho* gene to human longevity and survival rate, brain structure and function, psychiatric stress and treatment. For example, a larger-scale study of Italian population from young to old age showed that KL-VS heterozygous genotype was conducive to survival in the aged people through regulating α-Klotho protein transport and catalytic activity (21), as KL-VS heterozygous carriers had a higher level of serum α-Klotho than the non-carriers (23). Besides, KL-VS heterozygosity is associated with greater volume in the right dorsolateral prefrontal cortex (rDLPFC) and enhanced executive function in cognitively normal older adults (37), while G395A polymorphism is associated with reduced age-related cognitive impairment (50). One clinical study found a consistent correlation between SNP rs9315202 and psychiatric stress and cell aging, such as the strongest relationship between severity of PTSD and advanced epigenetic age in those carried two copies of the minor allele of rs9315202 (25). Besides, according to the forecast of the Enhancer DB website, the potential effect of rs9315202 on *Klotho* gene expression may be through transcription regulation, since it is located at a potential enhancer (enh51509) of the *Klotho* gene (26). In the treatment of late-life depression, patients with at least one SNP rs1207568 minor allele showed significant reduction of depressive symptoms (more than 50 percent reduction in Hamilton Rating Scale for Depression 21-items (HRSD-21) score) after 6 months of treatment, while the patients with rs9536314 homozygotes respond the treatment poorly (<10 percent reduction in HDRS-21 score) (22). We then selected KL-VS (rs9536314/rs9527025) and rs9315202 based on their known impact on the aging and brain function (23, 25, 50–52).

As only two participants were identified as KL-VS haplotype carriers, consistent with previous reports that this variant is very rare in Asian populations (53, 54), we excluded KL-VS from this investigation (Supplementary Tables 4, 5). These results suggest that the association between rs9315202 genotype and circulating α-Klotho in MDD patients is age-dependent. While knowledge of the physiological impact of Klotho polymorphisms on mental health is limited, one study showed that rs9315202 is associated with slower epigenetic aging in Caucasian (24), and higher level of circulating α-Klotho (23) as well as a positive effect on lifespan was seen in individuals who were heterozygous for the KL-VS allele (21, 27). In order to understand the function of the *Klotho* rs9315202 genotype in MDD, we divided all participants into two groups according to rs9315202 dominant model genotype (CC vs. CT + TT) and assessed the plasma levels of α-Klotho as well as some clinical features. We found a significantly lower frequency distribution of *Klotho* rs9315202 T carrier in the elderly first-episode MDD group than in the elderly controls (*P* = 0.039), while the elderly recurrent MDD group showed no difference in the distribution frequencies from the elderly controls (*P* = 0.347) (Table 2; Figure 2). However, between young MDD and controls, there is no difference in the rs9315202 dominant model distribution (Figure 2B). The elderly MDD T carriers had a higher plasma α-Klotho level than the age-matched MDD CC carriers, while the young MDD group failed to show an effect of rs9315202 genotype (Figure 3A). In addition, the elderly MDD T allele carriers had significantly lower HAMD scores than the elderly MDD CC carriers (Figure 3B). As a previous report suggested that the lower level of α-Klotho is associated with more severe depression-like symptoms in healthy people under chronic stress (18), we speculated that the *Klotho* polymorphism might affect the elderly MDD patients *via* reducing plasma α-Klotho levels. However, an effect of *Klotho* polymorphism on plasma α-Klotho levels and HAMD scores did not appear in the young MDD patients (Figures 3A,B). There was no significant correlation between plasma α-Klotho levels and HAMD scores in both young and elderly MDD patients (Figures 3C,D). Multiple linear regression was used to further analyze the factors affecting plasma protein in different groups (Table 5). *Klotho* polymorphism rs9315202 is a common factor affecting the plasma α-Klotho concentration in all participants, independent of age and sex. Together, our data suggest that *Klotho* polymorphism rs9315202 genotypes might be related to the plasma α-Klotho concentration in the elderly MDD patients. Only detecting plasma α-Klotho levels may not be specific to MDD, the combination of *Klotho* polymorphism and the expression of α-Klotho might be more useful for understanding the MDD in elderly.

There are several limitations in this study. First, our samples were grouped as young (30–40 years) and elderly (>66) and our data do not represent middle age (40–65 years) MDD. Second, there might be a potential effect of medication on the plasma α-Klotho levels in MDD patients in this study since we do not have a full treatment record for these patients from the biobank of Anding hospital and could not provide direct analysis of medication/treatment effect on plasma α-Klotho levels in MDD patients in this study, except having some discussion on potential medication involvement. These items should be addressed in the future investigations.

## Conclusion

The results of our study showed that only elderly MDD patients showed a decrease in plasma α-Klotho levels along with an increase in disease severity as well as an association with the number of rs9315202 T alleles, and not young MDD patients compared to age-matched controls. Our data suggest that circulating α-Klotho might be important in elderly MDD patients, particularly in carriers of the *Klotho* gene rs9315202 T allele.

## Data Availability Statement

The datasets presented in this study can be found in online repositories. The names of the repository/repositories and accession number(s) can be found below: NCBI SRA BioProject, Accession No: PRJNA691643.

## Ethics Statement

The study was approved by Ethics Committee of Beijing Anding Hospital and was in accordance with the latest version of the Declaration of Helsinki. The patients/participants provided written informed consent to participate in this study.

## Author Contributions

XG managed the literature searches, designed the study, undertook the sample processing, interpreted the statistical analyses, and wrote the first draft of the manuscript. ZS, YL, HX, and GM contributed to the assessment of plasma α-Klotho levels. JZ, YG, QD, GZ, and ML contributed to participant and clinical data administration. RL instructed in the study approach and supervised the statistical analyses and their interpretation. All authors have contributed to and approved the final manuscript.

## Funding

This research was supported by the National Natural Science Foundational of China (#9184910028 to RL) and the National Key Technology Research and Development Program of the Ministry of Science and Technology of China (#2020YFC2005300 to RL).

## Conflict of Interest

The authors declare that the research was conducted in the absence of any commercial or financial relationships that could be construed as a potential conflict of interest.

## Publisher's Note

All claims expressed in this article are solely those of the authors and do not necessarily represent those of their affiliated organizations, or those of the publisher, the editors and the reviewers. Any product that may be evaluated in this article, or claim that may be made by its manufacturer, is not guaranteed or endorsed by the publisher.
