# Supplementary material for: Reduced Plasma Levels of α-Klotho and Their Correlation With Klotho Polymorphisms in Elderly Patients With Major Depressive Disorders
Source: Front Psychiatry. 2021 Oct 13;12:682691. doi: 10.3389/fpsyt.2021.682691 (PMC8548667; doi:10.3389/fpsyt.2021.682691)
Supplement: Supplementary file 1 [file Data_Sheet_1.docx]

Supplementary Material

**Supplementary Table 1-5**

**Supplementary Table 1.**

Comparison of clinical data between healthy controls and MDD patients (mean ± SD).

| Characteristic | Healthy controls | MDD patients | *t* /*χ^2^* | *P* |
| --- | --- | --- | --- | --- |
| N | 112 | 144 |  |  |
| Female (%) | 60 (53.6) | 90 (62.5) | 2.070 | 0.150 |
| Age | 43.15 (13.64) | 51.19 (17.07) | - 4.187 | **<0.001** |
| Plasma α-Klotho levels, pg/ml | 808.660 (312.629) | 714.767 (313.075) | 2.382 | **0.018** |

**Supplementary Table 2.**

Pearson correlations between plasma α-Klotho levels and age in healthy controls and MDD patients.

| Groups | N | *r* | *P* |
| --- | --- | --- | --- |
| All subjects | 256 | - 0.564 | **<0.001** |
| Healthy controls | 112 | - 0.507 | **<0.001** |
| MDD patients | 144 | - 0.582 | **<0.001** |

**Supplementary Table 3.**

Genotyping and allele distributions of SNP rs9315202 in the young participants.

| Groups | rs9315202 genotype models | | | | | | | | | | | HWE |
| --- | --- | --- | --- | --- | --- | --- | --- | --- | --- | --- | --- | --- |
|  | Recessive | | Dominant | | Additive | | Genotype | | | Allele | |  |
|  | CC+CT | TT | CC | CT+TT | CC | TT | CC | CT | TT | C | T |  |
| Young HC | 66 | 14 | 27 | 53 | 27 | 14 | 27 | 39 | 14 | 93 | 67 | 1.00 |
| Young FEP | 27 | 8 | 9 | 26 | 9 | 8 | 9 | 18 | 8 | 36 | 34 | 0.98 |
| Young RP | 35 | 1 | 11 | 25 | 11 | 1 | 11 | 24 | 1 | 46 | 26 | 0.03 |
| *χ^2^* |  | | | | | | | | | | |  |
| *χ^2^* _Young FEP_ | 0.452 | | 0.731 | | 0.851 | | 0.903 | | | 0.887 | |  |
| *χ^2^* _Young RP_ | 4.780 | | 0.115 | | 3.048 | | 5.706 | | | 0.687 | |  |
| *P* |  | | | | | | | | | | |  |
| *P* _Young FEP_ | 0.502 | | 0.393 | | 0.356 | | 0.637 | | | 0.346 | |  |
| *P* _Young RP_ | 0.029 | | 0.735 | | 0.081 | | 0.058 | | | 0.407 | |  |

Note: HC, healthy controls; FEP, first-episode patients; RP, recurrent patients; data were analyzed by Pearson’s *χ^2^* test.

**Supplementary Table 4.**

Genotyping and allele distributions of SNP rs9536314 in total participants.

| Total participants (N = 256) | rs9536314 genotype models | | | | | | | | | | | HWE |
| --- | --- | --- | --- | --- | --- | --- | --- | --- | --- | --- | --- | --- |
|  | Recessive | | Dominant | | Additive | | Genotype | | | Allele | |  |
|  | TT+TG | GG | TT | TG+GG | TT | GG | TT | TG | GG | T | G |  |
|  | 256 | 0 | 254 | 2 | 254 | 0 | 254 | 2 | 0 | 510 | 2 | 1.00 |

**Supplementary Table 5.**

Genotyping and allele distributions of SNP rs9527025 in total participants.

| Total participants (N = 256) | rs9527025 genotype models | | | | | | | | | | | HWE |
| --- | --- | --- | --- | --- | --- | --- | --- | --- | --- | --- | --- | --- |
|  | Recessive | | Dominant | | Additive | | Genotype | | | Allele | |  |
|  | GG+GC | CC | GG | GC+CC | GG | CC | GG | GC | CC | G | C |  |
|  | 256 | 0 | 254 | 2 | 254 | 0 | 254 | 2 | 0 | 510 | 2 | 1.00 |
